# Anticipation in soccer: skilled players benefit from early pattern recognition in corner kick situations

**DOI:** 10.3389/fpsyg.2025.1631208

**Published:** 2025-08-06

**Authors:** Lovro Ivosevic, Tino Stöckel

**Affiliations:** Sport & Exercise Psychology Unit, Department of Sport Science, University of Rostock, Rostock, Germany

**Keywords:** corner kick, anticipation, expertise, occlusion, postural cues, pattern recognition

## Abstract

Anticipation is a crucial cognitive skill for decision making in life. Especially in sports, such as soccer, accurately anticipating the next play of the opposing team can have a huge impact on the result of a game or individual success in certain situations. This study investigates the differences between skilled players and less-skilled players in anticipating corner kicks by using temporal and spatial occlusion paradigms to figure out, which visual cues are utilized. We presented 171 video scenes of realistic corner kicks - stopped at three time points (150 ms before ball, at ball contact, 150 ms after ball contact) - to 23 skilled soccer players and 23 less-skilled players. Participants had to predict the horizontal landing position of the ball. To examine the effectiveness of different visual cues, participants saw the respective corner kick in full detail (control condition), or with either the kicker or the attacking players being blurred. Results showed that predictive accuracy increases over time and is higher in skilled players than in less-skilled players. Further, it appears that skilled players use information from the attacking players (i.e., pattern recognition) to estimate the landing position. Information of the kicker was not sufficient for neither the skilled players nor the less-skilled players. Our data strongly suggests, that expert soccer players´ advantage over less-skilled players in anticipating the landing position of corner kicks results from their ability to recognize patterns of play. Again, these findings highlight the importance of prolonged times of sport-specific deliberate practice for athletes’ predictive accuracy, and performance and success in general. Findings from this study could inform training regimes in soccer. By guiding players attention to useful visual cues (e.g., pattern recognition) while practicing set-pieces should help them to improve their anticipation skills. Future studies should explore these effects for situations like other set pieces or team-tactics.

## 1 Introduction

Anticipation—defined as the ability to recognize the outcome of a situation or the movement of an opponent before they occur—is a crucial cognitive skill in decision-making across various life situations, such as driving a car (Stahl et al., 2014), PC gaming (Nagorsky and Wiemeyer, 2020), and high-level sports (Williams et al., 2011). For example, in tennis, it is important to swiftly determine in which direction the opponent is attempting to hit the ball, because a player’s reaction time alone may not be sufficient to respond effectively. By processing relevant visual cues, such as the angle of the opponent’s racket, a skilled player is able to anticipate where the ball will land (Huys et al., 2009). While studies like these have examined anticipation skills in individual sport environments, anticipation is also a key criterion for success in team sports (Baker et al., 2003). Team sports are highly dynamic; thus, choosing the right action or reaction in a given situation to be successful depends not only on individual goals but also on the actions of the opposing team, one’s own teammates, and overall team tactics. Like in other team sports, soccer players must consider many factors in a short amount of time to respond appropriately.

Many studies have shown that differences exist between experienced and less experienced players in perceptual-cognitive tasks such as anticipation (Causer et al., 2017; Williams et al., 1993) or pattern recognition, which involves recalling previously seen and memorized patterns in team sports (Smeeton et al., 2004). Temporal and spatial occlusion paradigms have frequently been used to infer a superiority of skilled players over less-skilled athletes in such tasks (e.g., Causer et al., 2017; Abernethy and Russell, 1987). Temporal occlusion involves editing films by cutting out specific time periods or stopping scenes at various time points to obscure cues that players might use for cognitive processing (Farrow et al., 2005; Goulet et al., 1989). Usually, vision of the outcome of an action is blocked just before or after a visual cue that skilled observers can use to make better decisions. Spatial occlusion involves masking certain areas of a scene to obscure specific cues and determine their impact on decision-making (Dunton et al., 2020). Both paradigms are often combined. For example, Causer et al. (2017) used both to identify critical cues that distinguish skilled from less-skilled soccer goalkeepers in predicting penalty kick directions.

Penalties offer a unique context in soccer, where the goalkeeper’s success largely depends on interpreting the kicker’s visual information. These situations can be studied in lifelike ways. For example, Causer et al. (2017) used a camera angle and positioning mimicking a real goalkeeper’s view during a penalty kick. Skilled players’ superiority in utilizing visual cues ahead of decision-making has been linked to many hours of deliberate practice (Ericsson and Lehmann, 1996), suggesting that anticipation can be learned and improved.

Various theories explain why skilled players demonstrate superior anticipation skills. Using temporal occlusion, research shows that skilled players use cues more efficiently and earlier than less-skilled ones. For instance, Jones and Miles (1978) showed that experienced tennis coaches could predict the direction of a serve earlier than less-skilled players by testing occlusion at different timestamps. Their findings showed experts performed better in early occlusion conditions, suggesting skilled players rely on earlier visual cues. Farrow et al. (2005) confirmed these findings in a similar study.

Spatial occlusion research also suggests that skilled players are better at recognizing familiar patterns, aiding their anticipation (North et al., 2016). These players displayed better sports-specific memory and were more accurate in distinguishing familiar from novel sequences, likely due to a deeper understanding of relational player dynamics.

Gaze behavior is another important aspect. Research shows skilled players exhibit fewer, longer fixations than novices, indicating more efficient visual information extraction (Mann et al., 2007). Fixation location helps to identify decision-making cues, and fixation duration reflects processing demands. Skilled players often use visual anchor points to minimize eye movement, reducing cognitive load (Farrow and Abernethy, 2003; Williams and Davids, 1998).

Experienced performers also more effectively use situational probabilities to anticipate outcomes. For instance, Ward and Williams (2003) found elite soccer players leveraged contextual cues to better predict ball recipients. Similarly, Alain and Sarrazin (1990) found that expert squash players used situational knowledge to predict rally outcomes. Skilled athletes not only use situational cues but also draw on contextual knowledge of opponents’ action tendencies to enhance anticipation, demonstrating an ability to detect and adapt to consistent behavioral patterns. As highlighted in the review by Gredin et al. (2023), they can flexibly integrate prior information with unfolding visual cues underscoring the adaptable nature of expert perceptual decision-making.

To better summarize and contextualize the underlying cognitive processes that support anticipation and decision-making in elite soccer players, Habekost et al. (2024) propose a comprehensive three-stage model of cognition specifically adapted to the sport’s demands. The first stage—assessment of the current play situation—emphasizes how players actively gather and interpret a combination of visual cues, contextual information, and prior tactical knowledge, supported by attentional control and working memory. Skilled players efficiently integrate this information into coherent mental representations through domain-specific long-term working memory systems, enabling rapid pattern recognition without overloading their short-term memory (Ericsson and Kintsch, 1995; Habekost et al., 2024). In the second stage—action selection and execution—players select and perform motor responses by balancing speed and accuracy, often combining anticipatory strategies with reactive adjustments based on late but reliable perceptual cues (van der Kamp et al., 2018; Wright et al., 2013). This stage reflects the player’s ability to resolve uncertainty and act decisively under pressure, leveraging both procedural knowledge and executive control (Vestberg et al., 2012; Habekost et al., 2024). The third stage—assessment of outcome and feedback-based learning—involves evaluating the success of actions through sensory feedback and external information to refine perceptual and motor skills continuously. This iterative feedback loop contributes to the ongoing improvement of anticipatory skills and adaptive decision-making in the fast-paced environment of elite soccer (Habekost et al., 2024). By framing cognition as a dynamic, cyclical process involving perception, action, and learning, this model highlights the complexity and adaptability required for high-level performance.

To strengthen theoretical coherence and better contextualize the present research, it is important to emphasize that while visual anticipation and perceptual-cognitive skills have been examined in dynamic open-play situations, structured phases of play such as set pieces remain comparatively underexplored. Recent analyses, such as Clark et al. (2025), have highlighted the complexity and strategic importance of corner kicks at elite levels, noting the role of rehearsed routines, precise player positioning, and deceptive movement in influencing outcomes. Notably, Clark et al. (2025) report that over 70% of match results were influenced by successful corner kicks. Unlike penalties, these scenarios involve multiple players, synchronized tactics, and a broader range of visual demands on defenders and goalkeepers. Anticipation in such contexts likely draws not only on individual-level cues (e.g., gaze behavior, kicker biomechanics) but also on higher-order pattern recognition and team-level coordination. The current study addresses this gap by investigating anticipatory decision-making under temporal and spatial occlusion conditions during corner kicks—a more ecologically valid and tactically complex setting than those typically studied. In doing so, it builds on foundational work (e.g., Causer et al., 2017; Loffing and Hagemann, 2014) while extending the understanding of perceptual-cognitive mechanisms into a critical yet underrepresented area of soccer performance research.

The present study had two aims. First, to replicate findings that expert soccer players are more accurate in anticipating soccer-specific events than less-skilled players. As previous studies mostly addressed simpler scenarios (e.g., penalties; Causer et al., 2017), we examined corner kicks—more complex due to additional variables like multiple players and tactics. Anticipation could rely on cues from the kicker (e.g., hip/foot positioning; Loffing and Hagemann, 2014) or the attacking players’ formations. Using temporal occlusion, we stopped scenes 150 ms before, during, and after ball contact to test whether skilled players were more accurate. Based on past research suggesting that skilled players pick up cues earlier (Farrow et al., 2005; Jones and Miles, 1978), we expected greater accuracy, especially at earlier time points. We predicted that skilled players will demonstrate superior anticipation compared to less-skilled players across all temporal occlusion conditions. More specifically, skilled players are expected to show the greatest advantage when scenes are occluded 150 ms before ball contact, reflecting their enhanced use of early visual cues and pattern recognition processes (Farrow et al., 2005; Jones and Miles, 1978). We assume that this performance gap may decrease during and after ball contact, as more information becomes available to all participants.

Second, we sought to understand which visual cues skilled players use. Participants completed the same tasks under three spatial occlusion conditions: blurred kicker, blurred attackers, and control (no blurring). We hypothesized that skilled players perform best in the control condition with all information available, and that occluding either the kicker or the attackers will reduce performance in both groups. However, skilled players are expected to maintain superior accuracy due to their refined cue utilization and pattern recognition skills (Causer et al., 2017; Loffing and Hagemann, 2014; North et al., 2011; Savelsbergh et al., 2005). Furthermore, we predicted that blurring attackers will have a larger negative effect on anticipation before ball contact, when the visual information of the attacking players is crucial, whereas blurring the kicker will more strongly affect accuracy at or after ball contact, when specific movement cues dominate (Causer et al., 2017).

## 2 Methods

### 2.1 Participants

Forty-six male participants volunteered to take part in this study. Participants were assigned to either a skilled or a less-skilled group based on their playing experience. Recruitment took place from June 10th to June 19th 2024. For recruitment, we distributed a public call via an email distribution list from the University of Rostock. Key criterion to be deemed skilled was that the participant competitively played in a soccer club for at least ten seasons. No distinction was made regarding the playing level. The skilled group consisted of 23 participants (mean age = 22.25 years, SD = 1.74) who had been playing competitively for an average of 14.07 (SD = 3.33) seasons and were drawn from senior teams. The less-skilled group consisted of 23 participants (mean age = 22.99 years, SD = 3.12) with an average playing experience of 0.63 (SD = 1.41) seasons. The study was approved by the local institutional review board at the University of Rostock (A2025-0116) and conformed to the declaration of Helsinki. Participants provided written informed consent prior to participation and were free to withdraw from testing at any stage.

### 2.2 Experimental procedure

The experiment was designed to examine skilled soccer players’ and less-skilled participants’ ability to anticipate the trajectory and landing position of a corner kick. Therefore, video scenes of realistic corner kicks were stopped 150 ms before, at and 150 ms after ball contact, and participants had to predict the horizontal landing position of the ball in front of the goal (i.e., length of the corner kick). Moreover, the experiment was comprised of three different conditions that differed in the visual information that was provided to the participants to make their predictions. Participants could see the respective corner kick in full detail (control condition), or with either the kicker or the players that are about to receive the corner kick being blurred. Each condition consisted of 57 short video clips of corner kicks.

The film clips were presented using a 46-inch TV screen. Participants were sitting about 120 cm away from the screen. The experimenter was sitting on the right-hand side of the participant, out of eyesight. The whole experiment lasted about 30 min. After each scene participants were shown the last picture of the stopped scene as a still image with a grid over the image and they had to verbally tell the experimenter in which target field they think the ball would land. The experimenter noted the response. The end of the scene (i.e., actual landing position of the ball) was never shown to the participant to avoid learning during the experiment.

**Figure 1:**
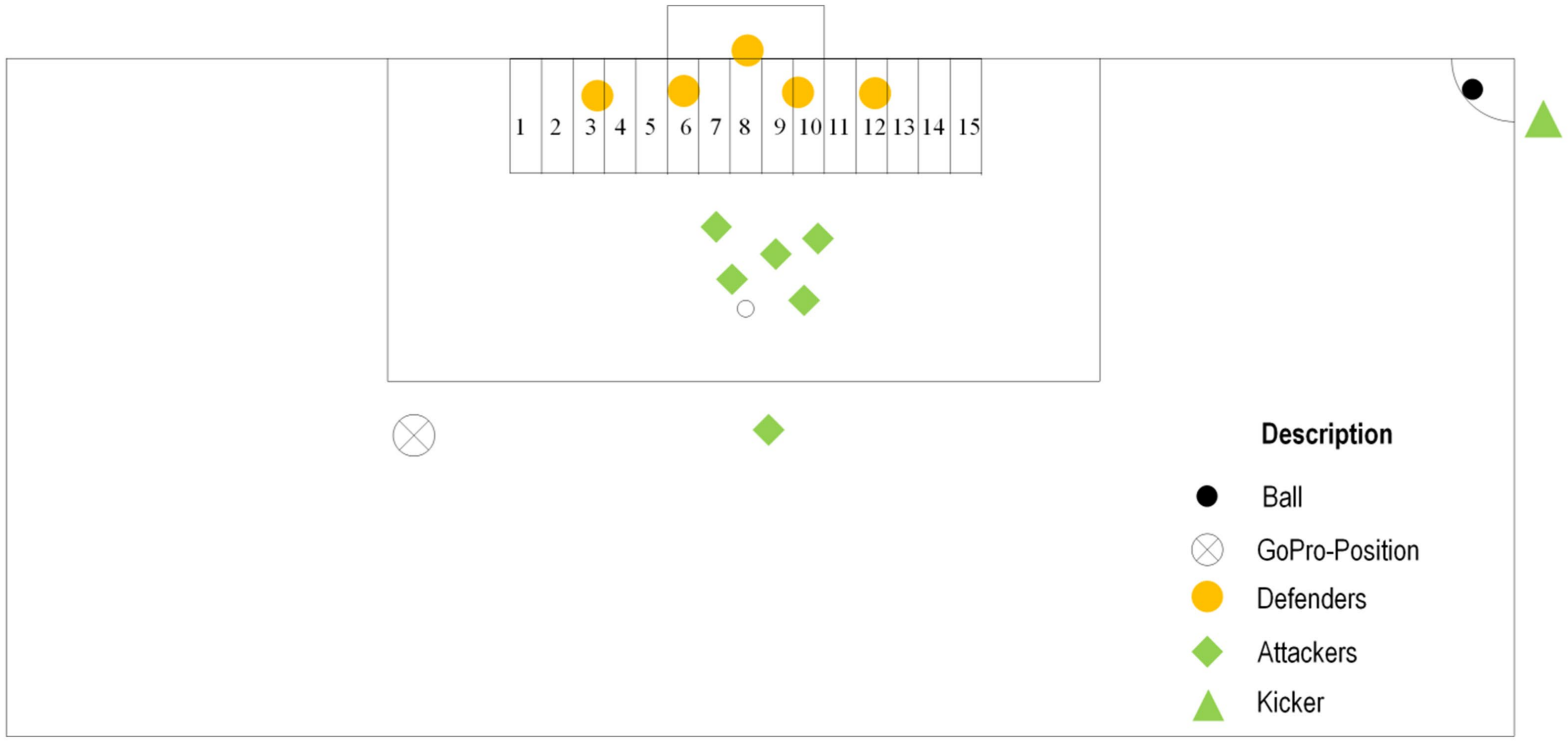
Experimental setup of the corner kick situation. The numbers mark the possible landing positions of the ball.

The test films were produced in cooperation with a German soccer club. To mimic real life scenarios of corner kicks during a match, there was an attacking team consisting of six players (excluding the kicker) and a defending team consisting of five players including the goalkeeper (see Figure 1). The recorded players were all part of a senior team playing at an amateur level. The corner kicks were recorded using a GoPro 7 positioned on the left-hand corner of the penalty box at about eye-level of an average young adult soccer player (174 cm) and about 19 meters away from the goal mimicking the perspective of another player of the attacking team. Three right-footed kickers were chosen based on the team’s usual corner kick takers. All of the kickers were asked to take nine corner kicks composed of three corner kicks toward the far post (far), three to the middle area of the goal (middle) and three toward the near post (near), resulting in a total of 27 corner kick scenes. The corner kick takers had to signal (as is common in soccer) their intended length by putting up one hand for near, none for middle and both for far, so the attacking team was informed about the intended length of the corner kick. The attacking team had the task to score a goal, while the defending team tried to defend their own goal. To make it even more relatable to a real match scenario, they did not have any further special assignment how to fulfill their tasks as attackers and defenders. They were simply asked to play the scenario as if they were playing an actual soccer match.

Out of the 27 corner kicks, those video clips were chosen that fulfilled the given requirement of hitting the appropriate length (5 short, 7 middle, 6 long) resulting in a total of 18 scenes. The recorded corner kicks were then digitally edited using the software Adobe Premiere Pro 7 to temporally and spatially occlude the scenes. Each scene was stopped at 150 ms before ball contact, during ball contact and 150 ms after ball contact (temporal occlusion), resulting in a total of 54 video clips of the 18 corner kicks. Further, the visual information of each scene was reduced (spatial occlusion) by blurring either the kicking player or the attacking players. This resulted in a total of 162 scenes in three visual information conditions (54 scenes in the control condition with all visual information available, 54 scenes with the kicker blurred [pattern condition] and 54 scenes with the attacking players blurred [kicker condition]). At the end of each scene a picture was extracted at the exact moment the clip ended (i.e., was stopped). Those pictures were then edited by laying a grid over the goal area so the whole goal including the edges of the five-meter area were covered. The grid was divided into 15 equally big target fields (150 cm in real life). Those fields were then numbered with 1 to 15 starting from behind the far post. The clips were then fed into the Be Gaze SMI 3.7.60 software (SensoMotoric Instruments Inc., Boston, USA). The video clips were presented in randomized order within each visual information condition (all information, kicker blurred, players blurred). Conditions were block-randomized between participants.

### 2.3 Data analysis

After viewing the video stimuli and the pictures with the overlaying grid with the target fields from 1 to 15, participants verbally indicated the target field which they assumed the ball would land in and an experimenter registered their answer. Participants’ responses (numbers between 1 and 15 for the respective target areas in front and around the goal; see Figure 1) were recorded for each scene and translated into an anticipation score (AS). The AS as a measure of predictive accuracy was defined as the absolute difference between the participants response (where they expected the ball to land in the respective scene) and the target field in which the ball actually landed. For example, if the participant assumes that the ball would land in target field number 10 (or number 2) and the ball actually lands in target field number 6, the AS for this response is 4. That means, a lower AS indicated a higher predictive accuracy. The AS was used as primary outcome measure for the participant’s ability to correctly predict the length of the corner kick. The AS was submitted to a group (skilled, less-skilled) x time (150 ms before ball contact, ball contact, 150 ms after ball contact) x condition (control condition, pattern condition, kicker condition) ANOVA. The alpha level for significance was set at 0.05. The Greenhouse–Geisser correction was applied when violations to sphericity were observed. For significant main or interaction effects Sidak adjusted simple comparisons were analyzed. Data are reported as mean (M) as well as mean difference (MD) along with the 95% confidence interval of the mean (95% CI). Partial eta-squared (*ƞ^2^*) is reported as measure of effect size.

## 3 Results

Raw anticipation scores for less-skilled players and skilled players in each of the three conditions 150 ms before ball contact, at ball contact and 150 ms after ball contact are shown in Table 1.

**Table 1:** Descriptive statistics for all conditions.

Group	Condition	Time	AS	SD
Less-skilled players	Control condition	−150 ms	4.089	0.153
		Ball contact	3.872	0.139
		+150 ms	3.797	0.177
	Kicker condition	−150 ms	4.147	0.129
		Ball contact	3.657	0.173
		+150 ms	3.553	0.110
	Pattern condition	−150 ms	4.114	0.159
		Ball contact	3.797	0.152
		+150 ms	3.551	0.146
Skilled players	Control condition	−150 ms	3.580	0.153
		Ball contact	3.529	0.139
		+150 ms	3.370	0.177
	Kicker condition	−150 ms	4.200	0.129
		Ball contact	3.652	0.173
		+150 ms	3.196	0.110
	Pattern condition	−150 ms	3.539	0.159
		Ball contact	3.570	0.152
		+150 ms	3.046	0.146

Mean anticipation scores (AS) and standard deviations (SD) for Less-skilled players and Skilled players in each of the three conditions 150 ms before (−150 ms) ball contact, at ball contact and 150 ms after ball contact (+150 ms). A lower anticipation score indicates a higher predictive accuracy.

The analysis showed no significant main effect for condition, *F* (2.88) = 1.458, *p* = 0.238, *ƞ^2^* = 0.032. However there was a main effect for time *F* (2.88) = 42.356, *p* < 0.001, *ƞ^2^* = 0.490, indicating that participants predictive accuracy significantly increased from 150 ms before to 150 ms after ball contact across all conditions and groups. Post-hoc simple comparisons confirmed that predictive accuracy was worst 150 ms before ball contact (*M* = 3.945, 95% CI [3.767, 4.123]), was significantly higher at ball contact (*p* < 0.001; *M* = 3.680, 95% CI [3.497, 3.863]) and best 150 ms after ball contact (*p* < 0.001; *M* = 3.419, 95% CI [3.247, 3.590]).

**Figure 2:**
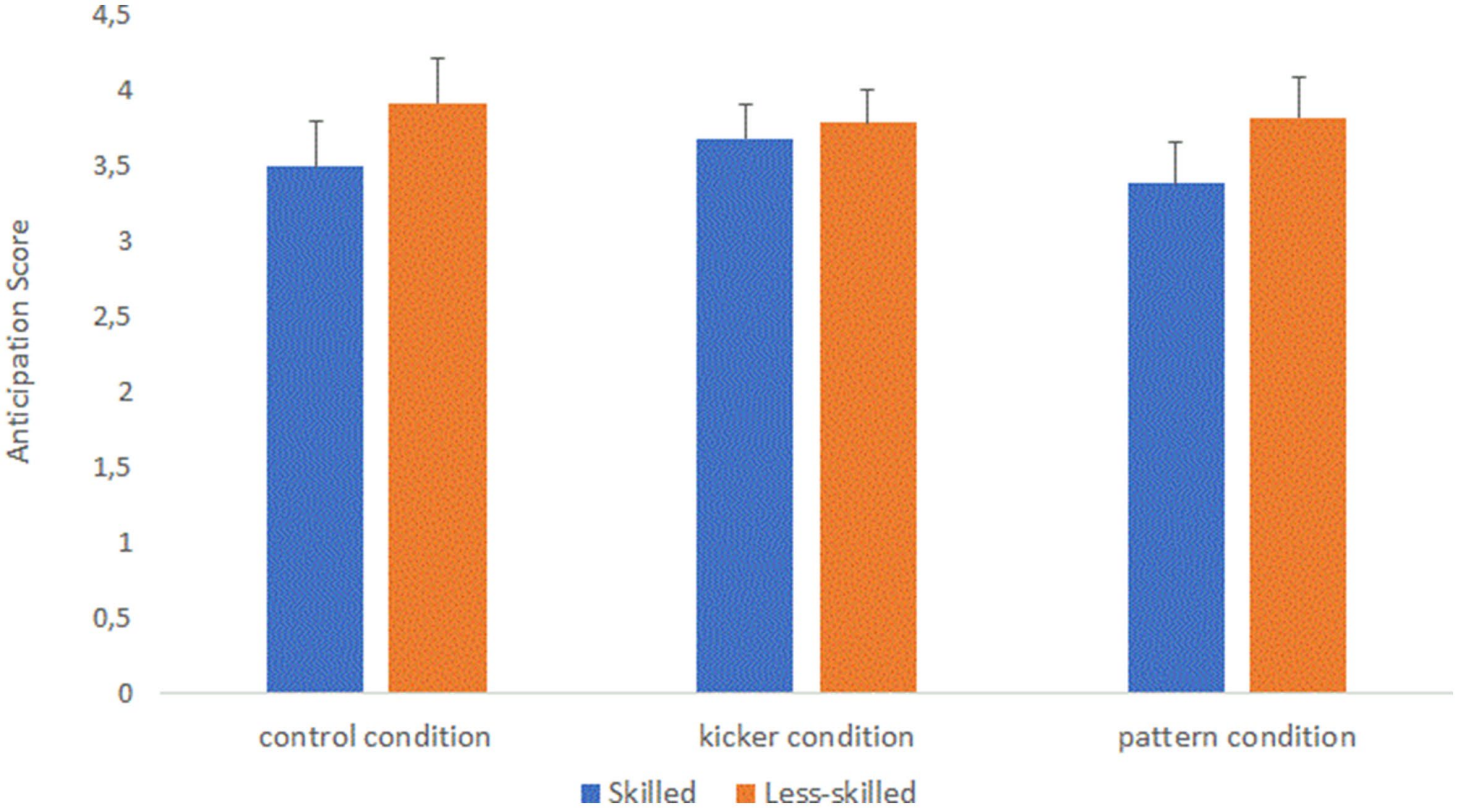
Interaction effect between group and condition. Error bars indicate 95% CI.

The analysis also revealed a significant main effect for group, *F* (1.44) = 3.876, *p* = 0.028 (one-tailed), *ƞ*2 = 0.081, confirming that skilled players (*M* = 3.520, 95% CI [3.287, 3.753]) are significantly better than less-skilled players (*M* = 3.842, 95% CI [3.609, 4.075]) in anticipating corner kicks. However, the significant interaction between group and condition, *F* (2.88) = 2.723, *p* = 0.036 (one-tailed), *ƞ*2 = 0.058, indicates that these expertise-dependent advantages differ between conditions (see Figure 2). *Post hoc* simple comparisons showed that skilled players outperformed the less-skilled participants in the control condition with all visual information (M_skilled_ = 3.493 vs. M_less-skilled_ = 3.919; *p* = 0.047; MD = 0.427, 95% CI [0.005, 0.848]) and in the condition in which the kicker was blurred (M_skilled_ = 3.385 vs. M_less-skilled_ = 3.820; *p* = 0.029; MD = 0.436, 95% CI [0.047, 0.824]), but not in the condition in which the attacking players were blurred (M_skilled_ = 3.683 vs. M_less-skilled_ = 3.786; *p* = 0.522; MD = 0.103, 95% CI [−0.219, 0.425]). Interestingly, predictive accuracy did not differ between conditions for the Less-skilled players (all *p*’s > 0.636), but Skilled players’ predictive accuracy was higher in the condition in which the kicker was blurred (*M* = 3.385, 95% CI [3.110, 3.660]) than in the condition in which the attacking players were blurred (*M* = 3.683, 95% CI [3.455, 3.910]) (*p* = 0.05).

**Figure 3:**
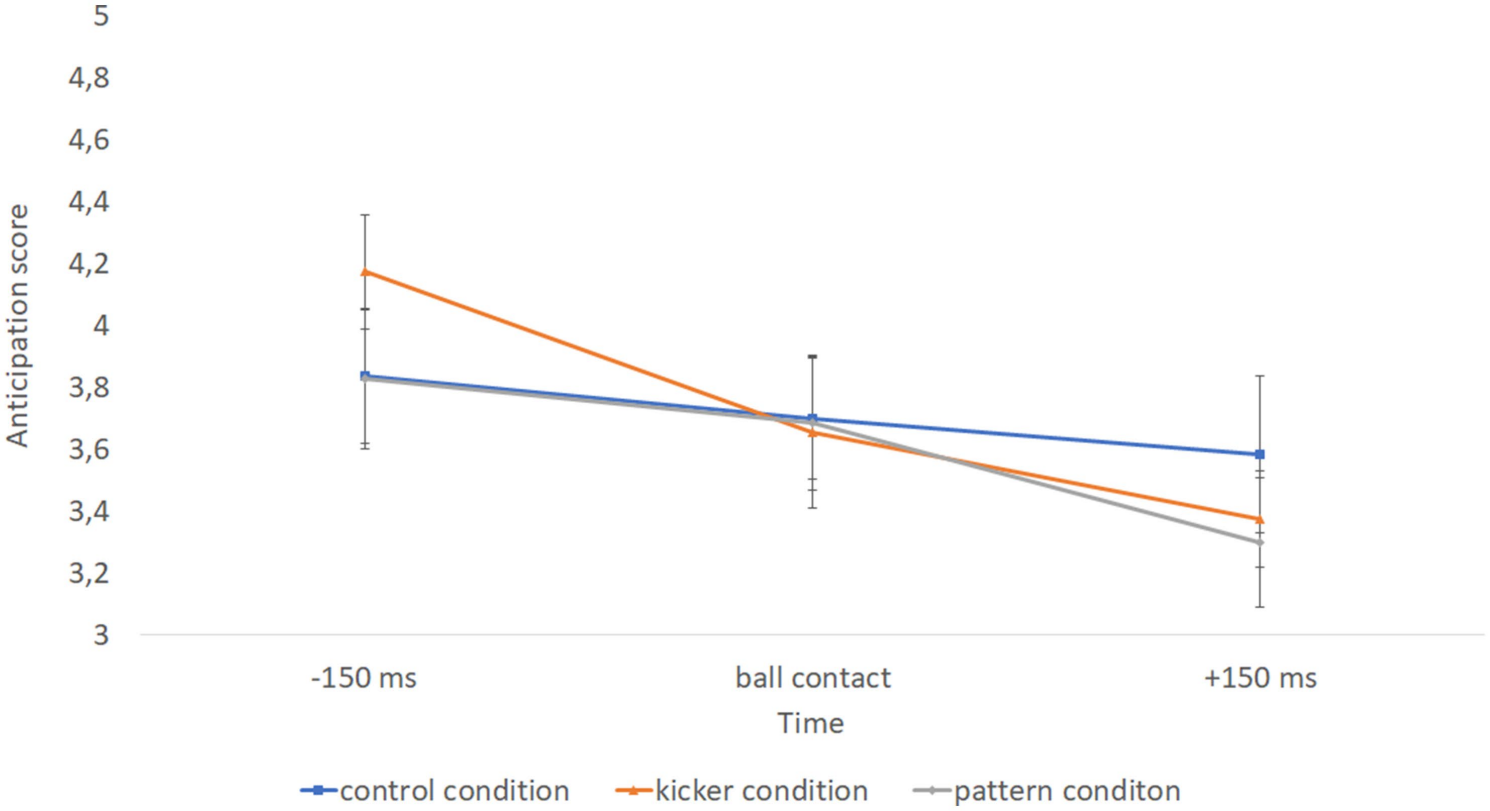
Interaction effect between time and condition. Error bars indicate 95% CI.

Our analysis also revealed a significant interaction between condition and time, *F.*(4.176) = 8.050, *p* < 0.001, *ƞ^2^* = 0.155, indicating that advantages for predictive accuracy over time differ between the three visual information conditions independent of the level of expertise (see Figure 3). Post hoc simple comparisons showed that 150 ms before ball contact predictive accuracy is worst in the condition in which the attacking players are blurred (*M* = 4.174, 95% CI [3.990, 4.358]) compared to the control condition (*M* = 3.835, 95% CI [3.616, 4.053]) and the condition in which the kicker is blurred (*M* = 3.826, 95% CI [3.599, 4.053]) (both *p* < 0.007). At ball contact, conditions do not differ at all (all *p*’s > 0.969). 150 ms after ball contact only the control condition (*M* = 3.583, 95% CI [3.330, 3.836]) differs from the condition in which the kicker is blurred (*M* = 3.298, 95% CI [3.090, 3.507]) (*p* = 0.015).

## 4 Discussion

The aim of this study was to examine the effect of expertise on the accuracy of anticipatory judgments of the landing position of (temporally and spatially occluded) corner kicks in soccer. In general, we hypothesized that skilled players (i.e., skilled players) are overall more accurate in predicting the accuracy of corner kicks due to better soccer-specific cognitive skills (e.g., anticipation skills, processing visual cues, or recognizing patterns of play; Loffing and Hagemann, 2014; Williams et al., 1993). Given the notion that skilled players pick up visual cues earlier than less-skilled players (Farrow et al., 2005; Jones and Miles, 1978), we predicted that skilled players should be more accurate than less-skilled players especially early during the decision-making process. With regard to the different visual information conditions, we assumed that players’ predictive accuracy is higher in the control condition than in blurred conditions with less information to base their predictions on. Yet, it remains unclear whether (and when during the decision-making process) information from the kicker or the attacking players helps skilled players to better anticipate the flight trajectory and landing position of the ball.

First, our data supports the notion that predictive accuracy increases over time as more information becomes available. Across both groups and all visual information conditions, predictive accuracy in anticipating the landing position of corner kicks significantly increased from 150 ms before ball contact to ball contact and again from ball contact to 150 ms after ball contact. This finding is well in line with previous research. For example, Causer et al. (2017) occluded penalty kicks in soccer at three different points in time (−160 ms, −80 ms, ball contact), and both groups (12 skilled and 12 less-skilled goalkeepers) were more accurate in predicting the direction and height of the penalties in later as compared to earlier occlusion conditions. Farrow et al. (2005) tested skilled and less-skilled tennis players in a similar way by showing both groups temporally occluded tennis serves from an opponent and let them predict the direction of the serve. In both groups, predictive accuracy significantly improved over the five occlusion conditions (900 ms before until 300 ms after racquet-ball contact). Back in 1990, Wright et al. (1990) already discovered for a volleyball-specific situation that participants (independent of their expertise) were better able to predict the passing direction of the setter (i.e., which hitter would get the ball) the later in the temporal sequence of a play they had to make their prediction.

Second, our data provides further evidence for the superiority of experts over less-skilled players in anticipating the outcome of sport-specific situations. In our study, skilled players outperformed less-skilled players in predicting the landing position of corner kicks across all conditions and occlusion points. Previous research already highlighted the importance of (sport-specific) cognitive skills for reaching higher levels in sports. For example, Roca et al. (2011) conducted a study in which participants were asked to predict (and react to) actions of their opponent in 11 versus 11 soccer scenarios from a defender’s perspective. They found that skilled soccer players were more accurate in predicting the intention of their opponents (and how to react accordingly) than less-skilled soccer players. Similarly, Helsen and Starkes (1999) claimed that skilled players have better anticipation skills than less-skilled players in their sport-specific domain. The skilled players were faster and also better able to find the right tactical solution than less-skilled players in a dynamic tactical decision-making task. In another study, Abernethy (1990) asked 16 skilled squash players and 20 less-skilled players to anticipate the direction and force of an opponent’s hit using test films. For all shown situations, skilled players were more accurate than less-skilled players in predicting the direction and force of the opponent’s hits in squash. That said, the superiority of skilled players over less-skilled athletes in anticipating the outcome of sport-specific situations appears to be a general marker of expertise (as a result of many years of deliberate practice and play in a specific field) that needs to be developed in order to be successful.

Another interesting finding of our study is that pattern recognition seems to be crucial for anticipating corner kick situations. Comparing the three visual information conditions in our study, our data suggests that expert soccer players use information from the attacking players to estimate the landing position of the ball. This finding is in line with the notion of skilled players’ refined ability to recognize patterns of play (e.g., North et al., 2016; Williams et al., 2006). Williams et al. (2006) managed to show the importance of pattern recognition in soccer by presenting familiar and unfamiliar film sequences of real-life game scenarios to skilled and less-skilled soccer players. Even when the scenes were only shown as point light patterns, the skilled players were able to recognize the scenes, showing that superficial visual information is not as important as the pattern of play itself. Surprisingly, our results also indicate that in the specific situation of a corner kick, pattern recognition is way more important than the information given by the kicker itself to estimate the landing position of the ball. Indeed, information from the kicker alone seems insufficient to predict the flight trajectory of the ball after a corner kick for both the skilled players and the less-skilled players. This contradicts previous research indicating that the kicker provides relevant visual information for anticipation and decision-making in soccer (e.g., Causer et al., 2017; Loffing and Hagemann, 2014; North et al., 2011; Feist et al., 2024). Causer et al. (2017) studied which cues helped skilled goalkeepers the most to successfully anticipate the direction and height of penalty kicks by occluding different regions of the kicker’s body, such as hips or legs. They found that skilled goalkeepers in soccer use visual cues from specific body regions to predict the direction and height of a penalty kick. Loffing and Hagemann (2014) showed that the angle of the run-up to the ball of the penalty taker is important to successfully predict the direction of penalty kicks. However, in those two studies, the participants had no other source of visual information to base their decision on. In our study, the participants could use either the information from the pattern of play of the attacking (and defending) players, or information from the kicker, or both. Since skilled players’ superiority over less-skilled players was only consistently proven for the pattern condition, in which the kicker information was blurred, it appears that the advantage of skilled players in anticipating the landing position of corner kicks results from their refined ability to recognize patterns of play. Based on these results, we assume that in complex situations of play, expert soccer players use the pattern of play as the most important visual source to base their decisions on. In complex situations, visual information from an opponent or teammate might even be too detailed, misleading, or distracting. In a qualitative study by Feist et al. (2024), they interviewed six skilled central defenders and seven experienced soccer coaches to explore how they experience their own anticipating behavior on the pitch and which cues are important for them for a quick and precise reaction. They found six factors, which seemed to be higher-order themes, of which one was sources of information. As sources of information, they then described the postural cues of the player with the ball as important in such a manner that if the attacking player with the ball looked up and his foot went back, they knew he was going to pass. Nevertheless, the direction of the pass they then predicted by the relative motion between the attacking players, so from that moment on, they relied on pattern recognition. North et al. (2011) also came to a similar conclusion by using point light and film displays of attacking scenes in soccer. Again, the skilled players were more accurate in anticipating the outcome of a certain situation, but the advantage was greater when using film displays. This indicates that postural cues were of some use for the skilled players but not for the less-skilled players. As the postural cues were not defined by North et al. (2011), it’s not entirely clear which cues were used by the skilled players. However, the corner kick in our study is already predestined in a sense of when the kicker will kick the ball, so the participants did not have to decide for themselves when the postural cues would appear. This does not necessarily contradict the research by North et al. (2011).

Finally, we found that skilled players are able to pick up useful visual cues earlier than less-skilled players in a corner kick situation. At these critical moments, skilled players likely rely on a combination of visual and tactical cues that are especially salient just before the corner kick is executed. Eye-tracking research in sports anticipation has shown that experts selectively fixate on task-relevant regions, such as the body parts of opponents or teammates and the spatial arrangement of players on the field, allowing them to extract predictive information efficiently (Mann et al., 2007; Williams et al., 2002). For example, Mann et al. (2007) demonstrated that expert soccer players focus their gaze earlier and longer on key cues such as the kicker’s hips and foot positioning, which are critical for predicting kick direction. Moreover, skilled players use their visual attention strategically to anticipate not only the kicker’s intentions but also team setup patterns and defensive alignment, integrating multiple sources of information simultaneously. This process is supported by the concept of the *quiet eye*, which describes how experts maintain prolonged, steady fixation on crucial visual cues immediately prior to action execution to optimize information processing (Vickers, 2007). Qualitative research (e.g., Feist et al., 2024) supports this view by highlighting how experts combine postural cues with an understanding of team dynamics to form rapid and accurate predictions. This suggests that the skilled players’ advantage at these time points is not merely based on isolated cues but reflects a holistic anticipatory process involving multi-cue integration, which unfolds in the final moments before ball contact. This interpretation aligns with the idea that expertise involves both perceptual adjustment to critical visual information and an advanced understanding of tactical patterns in dynamic play situations.

Supporting this, our data showed that pattern recognition is the most useful source for making early predictions (as compared to the kicker condition and to some extent to control condition) to have an advantage over less-skilled players. Since anticipatory performance in the control and pattern conditions are similar 150 ms before ball contact (but significantly better than the kicker condition), this could imply that pattern recognition is already sufficient to make an early accurate prediction for skilled players. Thus, it appears that skilled players did not find any more useful information in the postural cues by the kicker in addition to their pattern recognition at the first point of occlusion. This finding is partially supported by Causer et al. (2017), who showed that (by advanced cues utilization) expert goalkeepers outperformed the less-skilled players in predicting the direction of the penalty kick already at an occlusion point of 160 ms before the penalty kick was taken. However, anticipation in this study only relied on postural cues from the penalty taker, as there was no other source of visual information (like the pattern of attacking players in the present study) than the kicker himself. As previously mentioned, Williams et al. (2006) tested skilled and less-skilled soccer players by using real-life soccer scenes and edited point-light films and measured the response time and response accuracy. The skilled players were overall quicker and more accurate in their responses than the less-skilled players. Since they did not temporally occlude the scenes (i.e., scenes were always played until the end for every participant), one could argue that it does not necessarily imply an early recognition of pattern cues, but faster and quicker processing and decision-making. But this could also indicate that skilled players were able to outperform the less-skilled players because they recognized the pattern earlier while the scene was still played, which again would support our findings.

It is in our best interest as researchers to mention possible limitations for this study. First, with 46 male participants, the number of our tested subjects is rather low, and the statistical power of the data could be affected. With a greater number of participants, statistical power would be sufficient to draw meaningful conclusions from the three-way interaction, which is underpowered as of now. The fact that we have only tested male participants limits generalizability of our findings. Second, the corner kick scenarios filmed for the present study did not include professional players. The team used to generate the scenes played at a semi-professional level in the fourth German league, which could have an effect on the quality of the taken corner kicks – at least as compared to the highest competitive levels. It is possible that professional teams behave in a different manner in corner kick situations, like striking the ball with more precision or with more power, which could affect the trajectory of the ball and with that the outcome of the corner kick. Third, we used a constant angle and position from where the corner kick scenes were filmed (see Figure 1). This might benefit players who are used to this viewing angle due to their playing position, but other players might have a completely different viewing angle on the field and therefore may be at a disadvantage to predict the landing position. Future studies could use multiple angles and camera positions to cover a wider range of potential positions on the field. Lastly, while video-based paradigms offer a high degree of experimental control and reproducibility, they inherently limit ecological validity. Specifically, they lack the real-time motor responses, physical engagement, and psychological pressure present in actual gameplay. As such, the results may not fully capture the perception–action coupling and embodied decision-making processes critical to anticipatory performance in soccer. These aspects are central to expertise in dynamic sports environments and are challenging to replicate in passive viewing tasks.

In this study, we demonstrated anticipation as an important cognitive skill that skilled players in sports have over less-skilled players using the corner kick situation in soccer. Our findings highlight the importance of pattern recognition as a crucial skill for accurate prediction in highly complex play situations with attacking and defending players, where different visual information sources were available. These results can be used as practical implications for academy players or even professional players in soccer on how to act during a corner kick. Goalkeepers, for example, could train their anticipation performance by improving their pattern recognition. By implementing these findings, coaches could, for example, train corner kick situations in such a manner that the defense and the goalkeeper would have to react to the corner kick action by only observing the attacking players. Contrary, offenses could try and start using trick plays by design. For example, they could train team tactics in which they deceive the defense by occluding the pattern (e.g., all players start the attacking run from the same spot).

Coaches could also incorporate video-based learning environments where players review different corner kick scenarios and reflect on movement patterns, cues, and outcomes. These sessions might include freeze-frame tasks or prediction exercises to reinforce pattern recognition under varying conditions. For goalkeeper training specifically, drills could focus on identifying early visual cues from the kicker’s body orientation or initial movement patterns of attackers. Corner kick defense schemes could be designed to include variable attacker movements, requiring defenders to adapt in real-time rather than rely on fixed setups, thus simulating more realistic match conditions.

Another promising avenue for both practical application and research lies in the use of virtual reality (VR). VR technology enables the creation of immersive and interactive environments where spatial and temporal occlusion can be systematically manipulated. For instance, 360-degree film sequences of corner kicks can be presented through VR headsets, allowing players—particularly goalkeepers—to physically engage with the scenarios in real time. This approach reinstates critical elements of perception–action coupling and real-time decision-making, thereby enhancing ecological validity compared to traditional video-based lab tasks. It also enables the study and training of the quiet eye, a well-documented gaze behavior associated with improved decision accuracy and motor performance in sports (Vickers, 2007).

Future studies could leverage such immersive VR setups to investigate whether the anticipatory advantages observed in video-based tasks translate into real-life performance. Additionally, in-situ observations or field-based experiments could complement lab findings, helping to verify whether pattern recognition and anticipatory skills demonstrated under controlled conditions effectively transfer to live gameplay scenarios. Further research should extend this approach to other on-field situations in soccer to deepen understanding of the role and origins of anticipation and pattern recognition skills. Examining more complex team tactics or other set-piece situations could, for example, elucidate the broader impact of pattern recognition on players’ on-the-pitch anticipation. In this context, integrating gaze-tracking devices could shed light on how pattern recognition operates—specifically, which cues are processed, when, and how—and how gaze behavior, including the quiet eye, might be trained to enhance anticipatory skills (Vickers, 2007). Ultimately, this line of research should be taken into *in situ* field settings to validate effects in genuine soccer situations to offer novel pathways to improve anticipatory performance on the pitch.

## Data Availability

The raw data supporting the conclusions of this article will be made available by the authors, without undue reservation.
